# New Strategies for Stroke Therapy: Nanoencapsulated Neuroglobin

**DOI:** 10.3390/pharmaceutics14081737

**Published:** 2022-08-19

**Authors:** Santos Blanco, Esther Martínez-Lara, Eva Siles, María Ángeles Peinado

**Affiliations:** Department of Experimental Biology, Campus de Las Lagunillas s/n, University of Jaén, Building B3, 23071 Jaen, Spain

**Keywords:** stroke, neuroglobin, nanoformulation, ischemia

## Abstract

Stroke is a global health and socio-economic problem. However, no efficient preventive and/or palliative treatments have yet been found. Neuroglobin (Ngb) is an endogen neuroprotective protein, but it only exerts its beneficial action against stroke after increasing its basal levels. Therefore, its systemic administration appears to be an efficient therapy applicable to stroke and other neurodegenerative pathologies. Unfortunately, Ngb cannot cross the blood-brain barrier (BBB), making its direct pharmacological use unfeasible. Thus, the association of Ngb with a drug delivery system (DDS), such as nanoparticles (NPs), appears to be a good strategy for overcoming this handicap. NPs are a type of DDS which efficiently transport Ngb and increase its bioavailability in the infarcted area. Hence, we previously built hyaluronate NPS linked to Ngb (Ngb-NPs) as a therapeutic tool against stroke. This nanoformulation induced an improvement of the cerebral infarct prognosis. However, this innovative therapy is still in development, and a more in-depth study focusing on its long-lasting neuroprotectant and neuroregenerative capabilities is needed. In short, this review aims to update the state-of-the-art of stroke therapies based on Ngb, paying special attention to the use of nanotechnological drug-delivering tools.

## 1. Ischemic Stroke

Stroke is the second leading cause of death and the third most common long-term disability worldwide. While two-thirds of stroke deaths occur in less developed countries, stroke is the second most common cause of death in Europe, accounting for 650,000 deaths per year (OECD, 2020, https://www.oecd.org/health/health-data.htm, accessed on 25 May 2022). Moreover, stroke generates a large number of hospital admissions (70% of neurological admissions and the eighth leading cause of hospitalization), and is a leading cause of serious disability, with 15% to 30% of victims remaining permanently disabled. Consequently, the annual costs for stroke treatment and care in the EU are estimated at 27 billion euros per year, with 18.5 billion accounting for direct medical costs and 8.5 billion for indirect costs (e.g., loss of productivity). An additional 11.1 billion euros are estimated to account for informal care [1,2].

Although mortality rates have decreased in the last two decades, it is estimated that in 2035 the number of cases will increase by 39% in the EU and the total direct medical costs for stroke will triple (https://www.fesemi.org/publicaciones/otras/atlas-ictus-espana-, accessed on 25 May 2022); [2]. Hence, stroke has become a global social problem that threatens health and shortens life expectancy, affecting quality of life worldwide, especially when considering its relationship with COVID-19 sequelae [3].

There are two major types of stroke: (i) Hemorrhagic stroke (15–20%), that involves the rupture of a blood vessel causing bleeding in the brain, thus inhibiting oxygen and nutrients from reaching brain cells; and (ii) ischemic stroke, by far the most common type of stroke (80–85%), and caused by a blood clot or by the narrowing of a blood vessel thus leading to deprivation of oxygen and nutrients in an area of the brain. This last type of stroke produces infarcts, generally in the territory irrigated by the middle cerebral artery, affecting mainly the parietal cortex and striatum. The infarcted zone has a core and a penumbra, which is susceptible to recovery. The severity of damage depends both on the duration of the ischemia and on the level of reduction of the blood flow.

There are several ways to classify the severity of the infarct, but the best one consists in a combination of neuroimaging (e.g., magnetic resonance imaging (MRI) or computed tomography (CT)), and neurological evaluation using specific functional outcomes to identify behavioral deficits. The results of imaging and pathological parameters are correlated to the results of learning and memory/mood studies [4].

**Figure 1:**
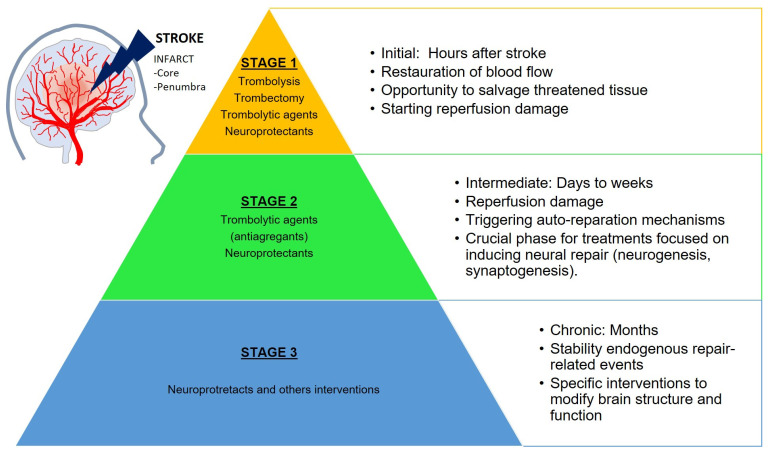
Temporal stages of the brain response towards stroke. Stage 1 occurs during the initial hours after the onset of stroke and represents an opportunity to salvage the threatened tissue, e.g., via reperfusion or neuroprotection. Stage 2 starts days to weeks following stroke and is crucial for treatments focused on the induction of the neural repair, as spontaneous neural repair may occur. Stage 3 represents a chronic phase where the brain is relatively stable with regards to endogenous repair-related events, although some modifications in brain structure and function are still possible with specific interventions.

The response of the brain to stroke can be roughly organized into three temporal stages (Figure 1):

- The first occurs during the initial hours after the onset of stroke and represents an opportunity to salvage the tissue, eliminate clots (thrombolysis, thrombectomy and thrombolytic treatments), and restore the normal blood flow. The harmful consequences of stroke (ischemic cascade) begin as soon as the ischemia occurs and continue during the reperfusion onset. The prognosis of the stroke depends on the severity of the ischemia in this stage and determines the outcomes of the following stages.
- The second phase lasts from days to weeks following the stroke. The reperfusion damage may continue (including neuronal death and inflammation) but spontaneous neural auto-reparation mechanisms also occur. This stage is crucial for treatments focused on the induction of neural repair (neurogenesis and synaptogenesis).
- The third stage is a chronic period lasting from months to years. The brain is relatively stable regarding the endogenous repair-related events, but modifications in brain structure and function are still possible with specific neuroprotectant interventions.

These three stages delineate distinct biological states and have clinical implications concerning the delivery of restorative therapies [5,6].

## 2. Neuronal Damage and Regeneration in Ischemic Stroke

**Figure 2:**
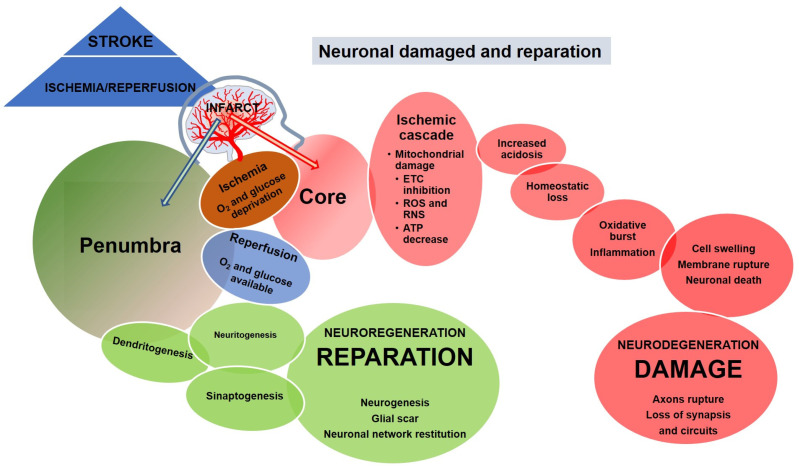
Ischemic stroke damage and repair mechanisms. Stroke triggers diverse mechanisms of damage and restoration during both the ischemic and reperfusion periods. The damage mechanisms mainly affect the core zone of the infarct. Conversely, repair mechanisms try to restore the damage zone and mainly occur in the penumbra of the infarct.

Neuronal injury in ischemic stroke is the consequence of a two-step process: (i) ischemia and (ii) reperfusion (I/R) (Figure 2). Ischemia, which involves depletion of oxygen and glucose, induces a series of major metabolic changes termed ischemic cascade. They include the inhibition of the electron transport chain (ETC), enhanced formation of reactive oxygen and nitrogen species (ROS, RNS), mitochondrial damage, energy depletion, acidosis, and loss of ionic homeostasis among others. These changes lead to cell swelling, membrane rupture, and eventually to neuronal death, usually in the ischemic core [7]. Reperfusion is associated with oxidative bursts and inflammation and is the cause of further damage [8,9]. The severity of the process depends on the duration of the ischemia and affects all cell types in the area, i.e., vascular cells, glia, and neurons. Neuronal deterioration and death imply the retraction and/or rupture of axons with loss of the corresponding neuronal circuits that, if not replaced, lead to functional damage that is difficult to recover [10].

While stroke injury results in a devastating set of damages, it also induces growth-related events in the perilesional zone that enable repair and the formation of new connections in the injured area. Adult neurogenesis occurs mainly at the subventricular zone of the walls of the lateral ventricle and the subgranular zone of the dentate gyrus. In pathological conditions such as stroke, increased neurogenesis has been reported in adult animal models and even in stroke patients [11]. Proliferating neural progenitor cells migrate to the injured striatum and cortex; however, most of them fail to survive and rewire the brain [12]. Hence, the enhancement of endogen mechanisms promoting the survival of new-born neurons may be a potential strategy for stroke repair. Axon regeneration during I/R is critical for the success of the re-establishment of damaged neurite networks and is driven predominantly by neuritogenesis-promoting signals. This process may launch soon after I/R (2–3 weeks poststroke) and is determined by the balance of driving and inhibitory signals and the glial scar formation [10,13]. Neuronal outgrowth enables the formation of functional axons and synapses in the brain, both over long and short distances, thus allowing the recruitment and formation of neuronal networks and/or restitution of those damaged by the stroke [14].

Hence, any protective action, either prophylactic or therapeutic, should not only aim to restore blood circulation in the affected area, but also to enhance those mechanisms that prevent cell and tissue damage and/or potentiate neural repair and neuroregeneration. In this way, controlling the production of free radicals, and therefore the oxidative damage, is a possible therapeutic target that has been investigated for years. Moreover, strategies addressed to reduce neuronal death and the consequent inflammatory signals, as well as to activate the processes of neurogenesis and synaptogenesis may also help in the recovery of lost neuronal circuits.

## 3. Current Treatments against Stroke

Effective treatments for ischemic stroke are limited, despite the plethora of attempts and therapies assayed (Table 1). Currently, intravenous thrombolysis with thrombolytic agents within 4.5 h after the onset of reperfusion, and thrombectomy combined with arterial thrombolysis within 6–24 h after the insult represent the two more effective strategies regarding the number of stroke survivors [15,16,17]. However, these strategies are not only highly dependent on the time elapsed until restoration of the blood supply, but may also have strict side effects and many risks. Therefore, it is important to seek for other treatments against the possible post-stroke complications, particularly considering that 40% of survivors are disabled after stroke due to the I/R injury [18]. Accordingly, in the last few years, different therapeutic approaches based on physical manipulations, new pharmaceutical entities, cell-based therapies as well as biomaterials have been proposed to offset the reperfusion damage and promote post-ischemic regeneration [19].

Specifically, treatments such as decompressive surgery or even hypothermia have been used [20], but they only slightly decrease the risk of becoming dependent after acute stroke [21]. Pharmacological therapies, such as aspirin, oxalate, glutamate, nitric oxide (NO) synthase inhibitors, antioxidants, or other drugs associated with the ischemic cascade have to some extent improved outcomes, but have not yielded the expected results [22]. One of the reasons for these failures is the lack of consideration of transport mechanisms at the blood–brain barrier (BBB) and neurovascular unit [23]. Hence, endogenous uptake transporters expressed at the BBB act as a gateway determinant of efficacious brain concentrations for centrally acting drugs; these may provide a great opportunity to advance stroke therapy via optimization of small molecule neuroprotective drug delivery to the brain [23,24].

In this context, the use of blood glutamate grabbers, such as riboflavin, have already been clinically validated, demonstrating their efficacy in patients with stroke [25,26]. Moreover, compounds such as statins, given their capability to low cholesterol levels, have been shown to be efficient for ischemic stroke [27]; nevertheless, the observation of some associated adverse effects such as intracranial hemorrhage, has also limited their use [28]. The possibility of interfering with many biological processes involved in the damage triggered by ischemic stroke and its comorbidities by using different types of miRNAs is also a promising tool recently proposed in the stroke therapy [29].

In any case, most of the pharmacological treatments tackle the BBB, a cellular interface that separates the central nervous system (CNS) from the periphery. The BBB regulates the trafficking of fluid, solutes, and cells between blood and the nervous tissue; it is integrated by endothelial cells, pericytes, glia, and neuronal cells. All these cells form the so-called “neurovascular unit”, contributing to the modulation of permeability after ischemic stroke [30]. Hence, the usefulness of any systemic drug for stroke therapy depends on its capacity to cross the BBB.

One of the most innovative pharmaceutical strategies to overcome the BBB is the use of different types of nanocarriers that may increase the lifespan of the associated therapeutic drugs in the bloodstream, enhancing their permeation through the BBB to effectively reach the ischemic brain site [31]. In addition, combination therapy with nanoparticles encapsulating neuroprotectants and tissue plasminogen activator (t-PA), a globally approved thrombolytic agent, has been demonstrated to extend the narrow therapeutic time window of t-PA [32]. There are many forms of nanocarriers, of different origin (organic, inorganic, and biological) that can be used for the delivery of imaging and therapeutic agents to the brain in different diseases and pathologies [33]. These nanoformulations, termed generically as drug delivery systems (DDS) include, among others, cell penetrating peptides (CPPs). CPPs are 5–30 aminoacid-long peptides with amphipathic or cationic motifs; they have mimetic sequences of viral vectors [34] and are able to cross the cell membranes [35], thus showing a great potential to deliver neurotherapeutics across the BBB for the treatment of ischemic brain injury [36]. Besides CPPs, the most recent DDS are cell membrane-derived vesicles (CMVs), such as exosomes and microvesicles [37]. Both of them are segregated from many cell types and show high biocompatibility and efficiency, although they are just beginning to be used in stroke [38]. More recently, biomimetic drug delivery systems have been designed using circulating cells ant their inherent capability to penetrate the ischemic brain [32].

Other popular noninvasive DDS are nanoparticles (NPs), whose most common design is based on lipids or polymers [39]. NPs are usually loaded with different drugs such as thrombolytics, metalloproteinases inhibitors (to regulate the BBB permeability), antioxidants, miRNA-126, or retinoic acid (to mitigate the inflammatory response), angiogenic factors and growth factors (to promote neurorepair), and even combinations of different molecules [40,41].

Liposomes and solid lipid nanoparticles (SLN) are NPs that can easily deliver different drugs throughout the organism [42]. Certainly, liposomes have been used in clinical practice as cargo systems to deliver chemotherapy agents, antibiotics, or antifungals, probing its effectiveness [42,43]. SLN show several advantages as DDS because they are made of physiological lipids, which have good biocompatibility, good size range (120–200 nm), and can be coated with a hydrophilic surface to avoid detection by the reticule-endothelial system. Regarding their ability to cross the BBB, their advantages as a strategy for brain drug delivery (longer circulation time, higher efficacy, reduced toxicity, site-specific targeted delivery via receptor-mediated transcytosis across brain capillary endothelial cells) overcome their limitations (smaller drug payload, storage, and administration stability issues) [44,45]. SLN can be also sterilized, obtaining stable formulations for as long as 3 years and whose release capacity lasts for several weeks [46]. Currently, and although this strategy is used in the clinic in cancer therapy, the scarce studies performed in stroke have failed at clinical trials while there is a lot of literature in culture and animal models [47,48,49]. In this regard, Karatas and collaborators (2009) [50] encapsulated a peptide inhibitor of the apoptotic protease caspase 3 into liposomal NPs coated with polyethylene-glycol. Their results showed a reduced infarct size and less neurological deficit in a rodent model of middle cerebral artery occlusion (MCAO). A similar strategy, followed by Reddy and Labhasetwar (2009) [51] using the antioxidant enzyme superoxide dismutase, improved the neurological outcome and reduced the infarct size in a rodent model of MCAO too. In rats, we have recently demonstrated the effectiveness of these nanocarriers to transport molecules like dopamine from blood to the brain [52].

Regarding polymeric NPs, they are also biocompatible and biodegradable, and have been used to deliver different drugs in I/R models of injury with good results. In this sense, Liu and collaborators [53] delivered cationic bovine serum albumin-conjugated tanshinone IIA PEGylated NPs into the ischemic rat brain, achieving a reduced infarcted volume and preventing neuronal apoptosis. Interestingly, we have conducted a series of studies with nanoencapsulated neuroglobin (Ngb) using sodium hyaluronate NPs in a stroke rat model of MCAO. The pharmaceutical preparation obtained (see later), was highly stable and biocompatible. In addition, it was able to transport the Ngb to the damaged neurons, thus demonstrating its neuroprotective effects against stroke [54,55,56].

## 4. Ngb: A Neuroprotective Protein against Stroke

Ngb is an oxygen-binding-heme protein of 17 kDa [57] with a monomeric hexacoordinate structure whose biochemical characterization is compatible with a gas sensor function upon hypoxic insults [58]. It is expressed in the CNS and peripheral nervous system (PNS), as well as in other multiple tissues; at cellular level, it is mainly localized in cytosol, inner wall of mitochondria, and nuclei [59].

Accumulating evidence has clearly demonstrated that Ngb acts as an endogenous neuroprotective molecule in numerous neurological diseases, including some hypoxic/ischemic (H/I) and oxidative stress-related insults [60]. This neuroprotective activity has been described in numerous publications from cultured neurons to animal models [61,62,63,64,65,66]. In fact, it has been proven that the expression of Ngb is increased in acute cerebral H/I in murine models [67,68], and even in human stroke patients [69,70]. This fact indicates that Ngb is a protein very sensitive to H/I. However, the expression of inducible Ngb should be at least tripled in order to achieve its neuroprotective potential [71,72]. In fact, the protective role of Ngb has been highly questioned when Ngb is expressed only at physiological level [73,74]. Interestingly, Ngb-overexpressing transgenic (Ngb-Tg) mice have been used to study the neuroprotective role of Ngb, not only in stroke but also in other neurological disorders, as reported by Shang and collaborators (2012) [75]. In stroke, Khan and collaborators (2006) [76] showed a reduction by 30% in the cerebral infarct size after MCAO using Ngb-Tg mice. In addition, the reduction of the size of the cerebral infarct was sustained up to 14 days after ischemia compared with wild type controls [77]. Moreover, knocking-down Ngb expression increased neuronal hypoxic injury in vitro and ischemic injury in vivo [77,78]. The intracerebral administration of a Ngb-expressing adeno-associated virus vector reduced the infarct size and improved functional outcomes in a MCAO model [79]. Consequently, throughout its short history of 20 years, Ngb has been proven to be a good therapeutic tool against stroke, but as long as its levels are increased in the infarcted area [80,81].

Several strategies have been used in animal model to increase Ngb in the damaged brain; some include genetic manipulations (i.e., Ngb-Tg mice) or the use of DDS (see below). Other options consist in up-regulating the expression of Ngb using molecules such as deferoxamine, hemin, or valproic acid, [68,69,82], although the side effects of these compounds, such as excessive iron chelation, seizures, pancreatic alterations, or bleeding, outweigh their benefits. Other alternatives include the use of natural compounds and synthetic steroids with estrogenic activity, as they all increase the expression of Ngb in cultured neural cells [62,83,84]. In a recent publication, Barreto and collaborators (2021) [85] have described the neuroprotective actions of estradiol on Ngb; this hormone upregulates and translocates Ngb into the mitochondria in order to sustain the neuronal and glial cell adaptation to injury of the CNS. In fact, sex differences in the steroid regulation of Ngb have been reported, particularly when it comes to assessing its neuroprotective role in reducing ischemic stroke damage [86].

## 5. Unraveling the Neuroprotective Action Mechanisms of Ngb

**Figure 3:**
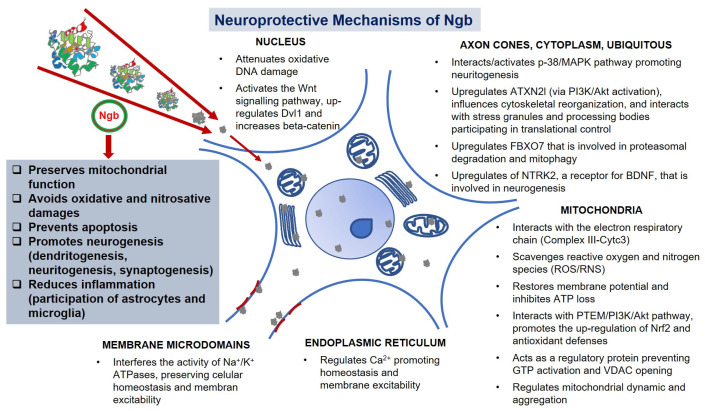
Neuroprotective mechanisms of Ngb. Neuroprotective protein Ngb enhances neuroregeneration by interfering in different processes and molecular pathways involved in neurogenesis and neuronal network restitution.

Despite the fact that the neuroprotective action of Ngb after the ischemic damage is well documented, the mechanisms underlying this role are not well known yet [60] (Figure 3).

One of the most prevalent hypotheses is the defensive role of Ngb against oxidative stress, probably through the scavenging of ROS and RNS [77,87,88,89]. The heme group of Ngb has reductase activity and may catalyze the reduction of NO_2_^−^ to NO, contributing to the cellular pool of this molecule [90]. However, Ngb may also react very rapidly with NO, yielding NO_3_^−^ by means of a heme-bound peroxynitrite intermediate, acting as an NO scavenger [91]. Besides this ability of Ngb to bind NO [92], some studies have shown parallelism between the distribution of Ngb and the neuronal nitric oxide synthase (nNOS) [93,94]. Moreover, NOS expression is increased in several tissues of Ngb-Tg mice [76]. These data may imply that in these neurons, NO could be an endogenous ligand for Ngb [81]. Indeed, after brain ischemia in mice, the infarcted areas show extensive presence of nitrated tyrosine residues [95]. Thus, the peroxynitrite scavenging action of Ngb may contribute to the neuronal survival following H/I episodes [96]. Therefore, interactions between Ngb and NO may result not only in NO scavenging, but also in nitrosative modification of proteins, including Ngb itself [97], which could alter the activity of these target proteins and trigger additional downstream neuroprotective pathways. These ligand-linked conformational changes based on reactions with O_2_ and NO have led Brunori and collaborators (2005) [91] to propose the involvement of Ngb in controlling the activation of a protective signaling mechanisms.

Interestingly, it has been reported that Ngb can directly interact with components of the respiratory chain (ETC), i.e., complex III, leading to a better mitochondrial functionality [98]. The neuroprotective action of Ngb against focal cerebral ischemia in Ngb-Tg mice can be due not only to a decrease in oxidative stress [99], but also to the preservation of the synthesis of ATP [77,89], or even to the attenuation of the oxidative damage to DNA [72,100]. Ngb may also interact with regulatory and signal transduction related proteins like PI3K or AMP-activated protein kinase (AMPK), involved in mitochondrial function and cell metabolism [101,102]. Using a therapeutic drug bioengineered with Ngb consisting of a manganese porphyrin reconstituted metal protein attached to Ngb (Mn-TAT-PTD-Ngb), Zhang et al. (2019) [103] obtained increased ROS scavenging ability of Ngb, thus boosting its neuroprotective activity. The authors describe that this artificial fusion protein, which has a more negative potential than Ngb alone, maintains the mitochondrial function by restoring its membrane potential and inhibits the loss of ATP in cell cultures. In addition, it prevented the activation of mitochondria-dependent apoptosis interacting with the PI3K/Akt pathway and promoted the up-regulation of Nrf2, which increased the expression of antioxidant defense enzymes such as SOD or CAT [103].

In this sense, different authors have reported the involvement of Ngb in the regulation of the PTEN/PI3K/Akt pathway. Specifically, an improvement of the neurological functions with a reduced infarct volume was achieved using a treatment with hemin to induce Ngb overexpression in a rat model of unilateral MCAO. Delving into the neuroprotective mechanisms of Ngb in this model, the activation of the PI3K/Akt signaling pathway has been pointed out [104]. Other studies, carried out in glioma cells, also revealed a strong association between Ngb and this same pathway, although, given that Ngb decreased the expression of Akt and consequently the apoptotic process in this model, the report concluded that Ngb may facilitate a malignant phenotype of glioma cells promoting proliferation and suppressing apoptosis [105].

The involvement of Ngb in the death pathways triggered by stroke may be also based on other molecular mechanisms. Specifically, the upregulation of Ngb expression in mice cortical neurons reduced the aperture of the mitochondrial permeability transition pore (MPTP), thus avoiding Cytc3 release after I/R [106]. Accordingly, it has been reported that the direct interaction between Cytc3 and Ngb could regulate the release of Cytc3 [107]. Other works described that Ngb can inhibit the cell death occurring after I/R, avoiding the elevation of cytoplasmic calcium through the modulation of channels in the endoplasmic reticulum [108]. Furthermore, Ngb can act as a guanine nucleotide dissociation inhibitor (GDI), inhibiting GDP dissociation from the Gα subunit of G-protein [74], or even through interactions with voltage-dependent anion channel proteins (VDAC) that form a channel through the outer mitochondrial membrane [81]. Mitochondrial dynamics, such as fission, fusion, mitophagy, or simply movements through cytoplasm are strongly impaired in ischemic stroke [109]. Upregulation of Ngb has also been related to this dynamic, preventing mitochondrial aggregation [101] and improving the cytoskeletal reorganization after I/R [110]. All these scientific reports undoubtedly lead to the conclusion that Ngb can play a central role in the improvement of the mitochondrial functions.

On the other hand, when Ngb is upregulated after neuronal injury, it shows other neuronal localizations and possible neuroprotective actions. In this sense, at a nuclear level, Ngb overexpression promotes neurogenesis in mice brains after ischemia; this effect was studied in cultured neural progenitor cells, and further validated in mice stroke models. In these studies, it has been suggested that the role of Ngb might be mediated through Dvl1 up-regulation, previous activation of the Wnt signaling pathway, leading to an increased nuclear localization of beta-catenin [111]. On the other hand, after transient global cerebral ischemia, Ngb is recruited in microdomains of the plasmatic membrane where it interferes with the activity of Na^+^/K^+^ ATPases, preserving cellular homeostasis and membrane excitability [112]. Ngb is also localized in axonal cones of the hypoxic neurons, either in cultures, animals, or humans, contributing to neuritogenesis via p38 interaction/activation [13]. Moreover, Ngb is also upregulated during neuronal development and localized in neurites, where it plays a critical role in neuritogenesis, interacting with PTEN and Akt differentially to activate the previously mentioned PI3K/Akt pathway [113]. Finally, Ngb has been detected in other nervous cells, such as microglia [113] or astrocytes [114], contributing to the regulation of processes like inflammation (for review see Barreto et al. 2021 [85]).

Proteomics and other omics studies can provide valuable information about the protective role of Ngb. In fact, monitoring the changes on key proteins not only helps to deepen the molecular bases of ischemia [115,116,117], but also the protective role of Ngb when it is delivered into the ischemic brain. Haines and collaborators (2012) [118] described that the protein interactome of Ngb is highly modified by hypoxia in murine neuronal cell lysates. These authors identified Ngb-interacting proteins that include partners consistent with antioxidative and antiapoptotic functions in neurodegenerative diseases, including stroke. Among the proteins identified, the authors determined a significant enrichment of intracellular protein with trafficking activities. Moreover, they found several signs to implicitly correlate hypoxic-Ngb changes with a massive regulation of the well-described synaptic plasticity after cerebral ischemic stroke [118].

In this regard, we have carried out proteomic studies in an animal model of MCAO, detecting a total of 219 proteins that significantly changed their expression after stroke and treatment with nanoencapsulate-Ngb [55]. Interestingly, our analysis shows a strong upregulation of ATXN2L (Ataxin-2-like protein), a protein involved in the assembly of stress granules (formed in response to diverse cellular stresses) and processing bodies (responsible for cytoplasmic mRNA processing). This protein, which also participates in cytoskeletal and mitochondrial reorganization, is activated via PI3K/Akt signaling [119]. Another strongly induced protein was FBXO7, a ubiquitin-protein ligase involved in the proteasomal degradation of target proteins related with mitophagy and negative regulation of oxidative stress-induced neuron death. Moreover, NTRK2, a receptor for BDNF involved in neurogenesis and plasticity pathways, was also upregulated in Ngb treated stroke animals [55].

Consequently, the neuroprotective role of Ngb, supported by its function as oxygen sensor, as well as its capacity to interact with other proteins involved in modulating reparative processes such as dendritogenesis, neuritogenesis, or synaptogenesis, allows Ngb to be assigned a central role in neuroregenerative therapy against stroke.

## 6. Exogenous Ngb in Stroke Therapy: Nanotechnological Tools

### 6.1. BBB: A Problem to Overcome

The easiest way to use Ngb as a therapeutic drug in stroke would be its direct pharmacological administration via intravenous (IV) injection; however, its molecular size and conformation prevent its crossing through the BBB [120,121]. Certainly, the BBB can be disrupted transiently [122,123] or continuously [124] during the acute injury phase after stroke, the extent and timing being dependent on several factors, such as age, genetic background, and gender. According to this, Ngb could easily reach the ischemic damage tissue under stroke; however, it has been shown that Ngb itself cannot efficiently cross the BBB in ischemic mice [125]. Consequently, the direct administration of systemic Ngb seems to be a more than questionable therapeutic strategy in stroke [126,127].

### 6.2. CPPs

A feasible strategy to overcome this problem, as previously pointed out, is the use of DDS that may deliver Ngb into the ischemic neurons. In this regard, exogenous Ngb has been linked to CPPs. This sort of DDS has been demonstrated to facilitate the entry of Ngb from blood into the damaged brain parenchyma [128]. Particularly, Cai et al. (2011) [125] successfully delivered Ngb macromolecules into the brain by attaching them to the 11-amino-acid HIV transactivator of transcription protein transduction domain (TAT-Ngb) in a mice model of MCAO. This CPP-protein fusion reduced the infarct size by about one third when it was injected 2 h before the artery occlusion; however, these same authors reported that the protection was negligible when the protein was applied just at the onset of reperfusion [125]. Since then, a new generation CPPs have been used as vehicles to transport many different drugs and molecules through the BBB in CNS diseases, including stroke [3], but so far, we have not found new attempts to use CPPs or other nanotechnological tools as media to facilitate the passage of Ngb through the BBB nor to convey it in in vivo models of stroke. However, CPPs and similar nanotechnological tools have been used in other neurodegenerative diseases [129].

### 6.3. Polymeric NPs

**Figure 4:**
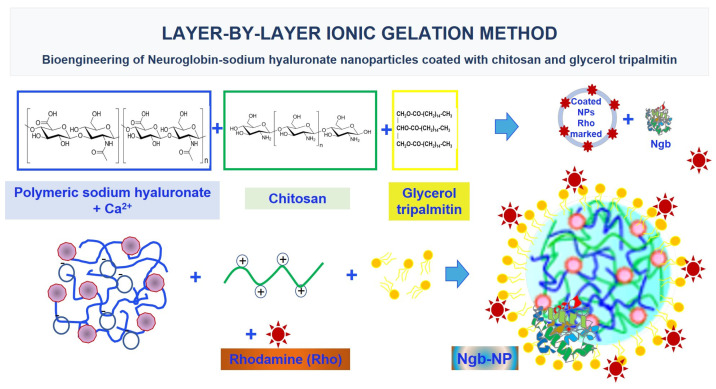
Layer-by-layer ionic gelation method: bioengineering of Ngb-sodium hyaluronate NPs coated with chitosan and glycerol tripalmitin. NPs have been constructed following layer-by-layer ionic gelation methods. These methods depend on ionic interactions between anionic chains of sodium hyaluronate and cationic charged polyions (e.g., calcium cations) as a cross linker. Then, these NPs can be coated with materials like chitosan or lipids, enhancing their specificity as carriers and providing better targeting. Finally, rat-recombinant Ngb has been attached to NPs (Ngb-NPs). These bioengineering Ngb-NPs are toxicity free and adequate in size and surface charge to deliver Ngb to the stroke damaged neurons.

Other strategies to transport Ngb through the BBB include their association to different DDS, such as biodegradable nano-vehicles. This is the case of the polymeric NPs, which are completely free of toxicity and can easily deliver the protein into the ischemic neurons. In this sense, polymer based nanocarriers can be labeled with different functional groups to monitor their specific targets [130]. They also protect the proteins from enzymatic degradation, may transport large amounts of proteins, and can circumvent the BBB and ultimately deliver the drug of interest to the brain [131], thus increasing the bioavailability of the cargo at the damaged tissue, as shown in different studies. More specifically, multifunctionalized NPs with high specificity towards cancer cells with endosomal escape capabilities [132], as well as biodegradable polymeric NPs that can accumulate in cells without activating the mononuclear phagocyte system [133] have been developed. In this line, we designed hyaluronate NPs by two ionic gelation methods, both coated with chitosan and glycerol tripalmitin to enhance their specificity and to provide better targeting and functionality [54,56] (Figure 4).

**Figure 5:**
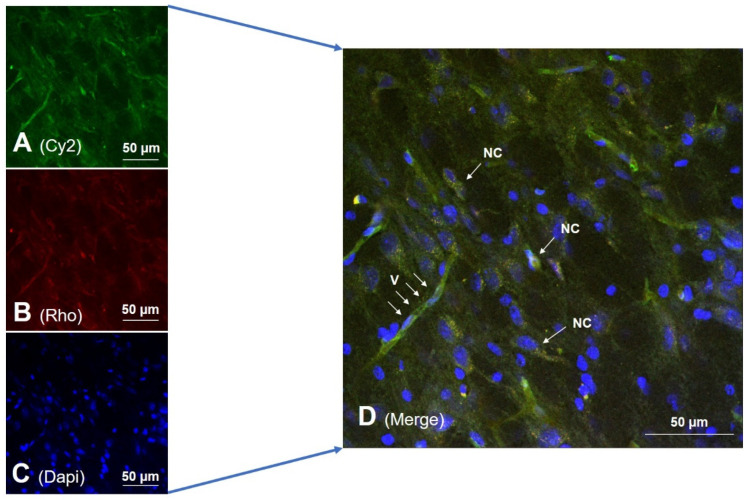
Ngb-NPs. Confocal microscopy images from the infarcted zone of MCAO animals taken 2 h after the systemic injection of the Ngb-NPs. Both the Ngb and NPs were colocalized in the vascular network and nervous cells of the injured brain parenchyma. (**A**) Cy2 green fluorescence represents Ngb. (**B**) Rhodamine red fluorescence detects NPs. (**C**) DAPI blue fluorescence marks cell nuclei. (**D**) Merge image showing the colocalization of Ngb and NPs inside vessels (V) and nervous cells (NC). Ngb-NPs appear in yellow, due to green and red merge.

After their formulation and characterization, our experimental purpose continued with the biosynthesis of rat-recombinant Ngb to complete the pharmaceutical preparation of nanoencapsulated Ngb-NPs. Then, Ngb-charged NPs were characterized by size, ζ-potential, encapsulation efficiently, in vitro release and kinetic liberation [56]. The NPs proved to be highly compatible with its pharmaceutical use, showing high efficiency as DDS, transporting the protective Ngb from blood to the damaged nervous cells. Using confocal microscopy and fluorescent labeling, both the NPs and the nanoencapsulated Ngb were colocalized in the vascular network and nervous cells of the injured brain parenchyma as soon as only 2 h after their systemic injection (Figure 5), and remained there after 24 h of reperfusion.

In our experiments, bioengineered Ngb-NPs were systemically injected in the MCAO animals immediately after the surgery at the onset of reperfusion. Their neuroprotective role against stroke was monitored during the next 24 h. These Ngb-NPs colocalized with the neuronal marker NeuN. They appeared grouped in clusters that showed the typical endosomal morphology. However, only a few of them colocalized with GFAP, a specific marker of astrocytes [55]. On the other hand, the encapsulated Ngb was able to exert its neuroprotective role as soon as 24 h after stroke; in this sense, MCAO animals treated with Ngb-NPs showed higher survival rates (up to 50%) and better neurological scores than the non-treated MCAO animals. Tissue damage also improved with the treatment, although we did not detect changes in infarct volumes or in the oxidative/nitrosative stresses values after 24 h of reperfusion [55].

### 6.4. New Challenges

Obviously, the next step should be to extend this study along the three temporal stroke stages to monitor the effects of Ngb-NPs in the long term. However, it will also be important to analyze the way in which Ngb-NPs reached the damaged tissue in the first hours of the reperfusion period. In this sense, and considering the different pathways by which molecules and ions cross the BBB to reach neurons [30], and the physico-chemical nature of our nanoformulation complex [54,56], we could discuss two important matters: (i) the way in which the Ngb-NPs cross the BBB and reach the neuron endosomal compartment and (ii), once there, what is the escape mechanism of Ngb-NPs so that they can reach their intracellular targets.

## 7. Delving into the Mechanisms of the Delivery of Ngb-NPs

Given the optimum size of the Ngb-NPs and its decoration with chitosan and glycerol tripalmitin, it could be thought that they might cross the BBB and reach the neuron endosomal compartment via several cell-penetrability mechanisms, including macropinocytosis, adsorptive-mediated transcytosis, or tight junctions opening at the neurovascular unit level of the BBB [134]. Moreover, the presence of sodium-hyaluronate in the composition of the Ngb-NPs could also suggest a possible receptor-mediated-endocytosis pathway. In this sense, it is known that cells have hyaluronate receptors, CD44 and the receptor for hyaluronan-mediated motility (RHAMM) being the most common. Nevertheless, both are scarcely represented in the nervous cells of the vascular unit of the BBB [135] and although they increase after stroke, they are only found to be upregulated in inflammatory cells (macrophages-microglia) [136]. In this sense, polymeric NPs with hyaluronic acid in their composition have been engineered to transport doxorubicin to cancer cells, suggesting that they bind to CD44 receptors, highly expressed in these cells, to cross the plasma membrane [132]. Anyway, the receptor-endocytic pathway via CD44 and RHAMM receptors is questionable in neurons, and not only by their above-mentioned absence in neural cells, but also because these receptors are involved in different cellular functions other than endocytosis [135]. In addition, in our experiments, the Ngb-NPs were located almost exclusively in the endosomal compartment of the neurons 24 h after their injection [55]. This selective capture of the NPs by neurons is also an issue that should be considered in order to get a better understanding of the drug delivery strategies either for Ngb or for other macromolecules.

A second matter, concerning the intraneuronal traffic of the Ngb-NPs, refers to the endosomal escape mechanism. Knowledge about this mechanism comes mainly from experiments in cultured tumor cells (for review see Donahue et al. 2019 [137]) which have provided important information about the endosomal trapping dynamics. Interestingly, the endosomal compartments undergo a series of transformation stages, passing from early to late endosomes, and these stages are accompanied by changes in their intra-vesicular pH that acidifies over time. Undoubtedly, these changes must affect the cargo escape process and any nanoformulation or therapeutic agent should be appropriately designed to avoid the endosomal entrapment and later lysosomal degradation at the right time [138]. Accordingly, different endosomal escape strategies have been described; one of the most known consists of a mechanism of membrane-disruption by surface modifications, as in the case of CPPs or lipids, that favors their fusion with the endosomal membrane. This mechanism, that in the case of CPPs has been named “the proton sponge effect”, has been attributed to cationic surface modifications which could induce osmotic swelling, membrane disruption, and finally the escape of both the cargo and the nanocarrier together. Other strategies are based on the liberation of the cargo from its nanocarrier inside the endosome, either due to pH changes or to cleavable enzymes. Both strategies allow the free cargo to cross the endosomal membrane and reach their subcellular targets. Possibly, and given the polymeric nature of our Ngb-NPs, they could interact with the endosomal luminal membrane through charge–charge, leading to local membrane destabilization and permeability. This punctual destabilization/disruption process would avoid the complete lysis of the endosome, suggested by the “proton sponge” hypothesis [139], thereby maintaining the endolysosomal compartment intact during and after the escape.

Although further research will be needed to elucidate all these questions, the fact that a nanopharmaceutical preparation of systemic Ngb capable of reaching the neurons is already available is an important step in stroke therapy and possibly in the therapy of other neurodegenerative diseases.

## 8. Conclusions

Ngb is an oxygen sensor protein with neuroprotective activity against many brain injuries, including neurodegeneration, toxicity, nutrient deprivation, and ischemia. Although Ngb is an endogenous protein, its protective effects seem to rely on its overexpression or increase in the damaged tissue. Thereby, the injection of exogenous Ngb into the blood stream could be a good therapeutic option in some neurological diseases, including stroke. However, Ngb cannot cross the BBB itself, so therapeutic approaches using nanotechnological tools have arisen as promising alternatives. In fact, the use of nanoparticles as a contrast agent or as drug carriers for a specific target are some of the most common approaches developed in nanomedicine for stroke [140]. In addition, these DDS not only exert a freight function, but they also protect their cargo from immune threats, leading Ngb to their final targets in the ischemic damaged neurons.

More specifically, polymeric sodium hyaluronate NPs fulfill all the characteristics of DDS, as they protect exogenous Ngb from the endothelial system in blood and facilitate the passage of Ngb through the neurovascular unit of the BBB. This path depends on numerous cellular processes such as membrane permeability, cytoskeletal motility, endosomal and mitochondrial dynamics, and homeostatic energy or ion regulation, among others. In fact, the bioengineered Ngb-NPs assayed by our group seemed to overcome all these processes reaching their intracellular targets as soon as 2 h after injection. What is more, the Ngb delivered this way exerts its neuroprotective role in stroke for as long as 24 h after the onset of reperfusion.

Although these nanotherapeutic strategies against stroke are very promising, further research is needed. Hence, we have identified the following points for further investigation: (i) More in-depth study of the neuroprotective mechanisms exerted by Ngb in the ischemic tissue. (ii) Comprehensive identification of the many pathways and processes involved in the long journey that the Ngb-NPs run from the blood to their final destinations in the ischemic damaged neurons. (iii) Detailed follow up of the evolution of this nanotechnological treatment along the different post-stroke stages.

Thereafter, the therapeutic horizon of these nanotechnological tools is hopeful. Notwithstanding, further scientific findings are needed in order to optimize these therapies in stroke.

## Figures and Tables

**Table 1:** Current treatments against stroke.

Therapy	Advantages	Disadvantages
Intravenous thrombolysis	Effective 4.5 h after the onset of reperfusion	Narrow therapeutic window Strict side effects Many risks
Thrombectomy combined with arterial thrombolysis	Effective 6–24 h after the insult	Narrow therapeutic window Strict side effects Many risks
Decompressive surgery	Reduce mortality and improve functional outcomes	Slightly decrease the risk of becoming dependent after acute stroke
Hypothermia	Reduce mortality and improve functional outcomes	Slightly decrease the risk of becoming dependent after acute stroke
Several pharmacological therapies: Aspirin Oxalate Glutamate Nitric oxide synthase inhibitors Antioxidants Other drugs associated with the ischemic cascade	Improved the outcomes only to some extent	More research needed Have not yielded the expected results
Blood glutamate grabbers	Already clinically validated	Relatively novel approach: more research needed
Statins	Low cholesterol levels	Intracranial hemorrhage
miRNAs	Can interfere with many biological processes	Relatively novel approach: more research needed
**Drug delivery systems (DDS)**		
Cell penetrating peptides (CPPs)	Ability to cross the cell membranes	Complex technical approach
Cell membrane-derived vesicles (CMVs): Exosomes Microvesicles	High biocompatibility and efficiency	Relatively novel approach: more research needed
NPs:	Can be loaded with a broad range of drugs	
Liposomes	Proven effectiveness delivering chemotherapy agents, antibiotics, or anti-fungals	Scarcity of studies in clinical trials, most literature in culture and animal models
Solid lipid nanoparticles (SLN)	Good biocompatibility Good size range Can be sterilized Stable formulations Can avoid its detection by the reticule-endothelial system	Scarcity of studies in clinical trials, most literature in culture and animal models
Polymeric NPs	Biocompatible Biodegradable Success in delivering Ngb	Scarcity of studies in clinical trials, most literature in culture and animal models

## Data Availability

Not applicable.

## References

[1] Alvarez-Sabín J., Quintana M., Masjuan J., Oliva-Moreno J., Mar J., Gonzalez-Rojas N., Becerra V., Torres C., Yebenes M., CONOCES Investigators Group (2017). Economic impact of patients admitted to stroke units in Spain. Eur. J. Health Econ..

[2] Rajsic S., Gothe H., Borba H.H., Sroczynski G., Vujicic J., Toell T., Siebert U. (2019). Economic burden of stroke: A systematic review on post-stroke care. Eur. J. Health Econ..

[3] Zhang S., Zhang J., Wang C., Chen X., Zhao X., Jing H., Liu H., Li Z., Wang L., Shi J. (2021). COVID-19 and ischemic stroke: Mechanisms of hypercoagulability (Review). Int. J. Mol. Med..

[4] Pérez-Asensio F.J., Hurtado O., Burguete M.C., Moro M.A., Salom J.B., Lizasoain I., Torregrosa G., Leza J.C., Alborch E., Castillo J. (2005). Inhibition of iNOS activity by 1400W decreases glutamate release and ameliorates stroke outcome after experimental ischemia. Neurobiol. Dis..

[5] Cramer S.C. (2018). Treatments to Promote Neural Repair after Stroke. J. Stroke.

[6] Wu Q., Yan R., Sun J. (2020). Probing the drug delivery strategies in ischemic stroke therapy. Drug Deliv..

[7] Auriel E., Bornstein N. (2010). Neuroprotection in acute ischemic stroke—current status. J. Cell. Mol. Med..

[8] Di Domenico F., Casalena G., Jia J., Sultana R., Barone E., Cai J., Pierce W.M., Cini C., Mancuso C., Perluigi M. (2012). Sex differences in brain proteomes of neuron-specific STAT3-null mice after cerebral ischemia/reperfusion. J. Neurochem..

[9] Wu M.-Y., Yiang G.-T., Liao W.-T., Tsai A.P.Y., Cheng Y.-L., Cheng P.-W., Li C.-Y., Li C.J. (2018). Current Mechanistic Concepts in Ischemia and Reperfusion Injury. Cell. Physiol. Biochem..

[10] Carmichael S.T., Kathirvelu B., Schweppe C.A., Nie E. (2017). Molecular, cellular and functional events in axonal sprouting after stroke. Exp. Neurol..

[11] Koh S.-H., Park H.-H. (2017). Neurogenesis in Stroke Recovery. Transl. Stroke Res..

[12] Casares-Crespo L., Calatayud-Baselga I., García-Corzo L., Mira H. (2018). On the Role of Basal Autophagy in Adult Neural Stem Cells and Neurogenesis. Front. Cell. Neurosci..

[13] Xiong X.X., Pan F., Chen R.Q., Hu D.X., Qiu X.Y., Li C.Y., Xie X.Q., Tian B., Chen X.Q. (2018). Neuroglobin boosts axon regeneration during ischemic reperfusion via p38 binding and activation depending on oxygen signal. Cell Death Dis..

[14] Hermann D.M., Chopp M. (2014). Promoting Neurological Recovery in the Post-Acute Stroke Phase: Benefits and Challenges. Eur. Neurol..

[15] Beslow L.A., Smith S.E., Vossough A., Licht D.J., Kasner S.E., Favilla C., Halperin A.R., Gordon D.M., Jones C.I., Cucchiara A.J. (2011). Hemorrhagic Transformation of Childhood Arterial Ischemic Stroke. Stroke.

[16] Terruso V., D’Amelio M., Di Benedetto N., Lupo I., Saia V., Famoso G., Mazzola M.A., Aridon P., Sarno C., Ragonese P. (2009). Frequency and Determinants for Hemorrhagic Transformation of Cerebral Infarction. Neuroepidemiology.

[17] Van Den Berg L.A., Dijkgraaf M.G.W., Berkhemer O.A., Fransen P.S.S., Beumer D., Lingsma H.F., Majoie C.B.L.M., Dippel D.W.J., Van Der Lugt A., Van Oostenbrugge R.J. (2017). Two-Year Outcome after Endovascular Treatment for Acute Ischemic Stroke. N. Engl. J. Med..

[18] Bustamante A., García-Berrocoso T., Rodriguez N., Llombart V., Ribo M., Molina C., Montaner J. (2016). Ischemic stroke outcome: A review of the influence of post-stroke complications within the different scenarios of stroke care. Eur. J. Intern. Med..

[19] Rajkovic O., Potjewyd G., Pinteaux E. (2018). Regenerative Medicine Therapies for Targeting Neuroinflammation after Stroke. Front. Neurol..

[20] Van Der Worp H.B., Macleod M.R., Bath P., Demotes J., Durand-Zaleski I., Gebhardt B., Gluud C., Kollmar R., Krieger D.W., Lees K.R. (2014). EuroHYP-1: European Multicenter, Randomized, Phase III Clinical Trial of Therapeutic Hypothermia plus Best Medical Treatment vs. Best Medical Treatment Alone for Acute Ischemic Stroke. Int. J. Stroke.

[21] A Donnan G., Davis S.M. (2008). Breaking the 3 h barrier for treatment of acute ischaemic stroke. Lancet Neurol..

[22] Broussalis E., Killer M., McCoy M., Harrer A., Trinka E., Kraus J. (2012). Current therapies in ischemic stroke. Part A. Recent developments in acute stroke treatment and in stroke prevention. Drug Discov. Today.

[23] Ronaldson P.T., Davis T.P. (2022). Transport Mechanisms at the Blood–Brain Barrier and in Cellular Compartments of the Neurovascular Unit: Focus on CNS Delivery of Small Molecule Drugs. Pharmaceutics.

[24] Williams E.I., Betterton R.D., Davis T.P., Ronaldson P.T. (2020). Transporter-Mediated Delivery of Small Molecule Drugs to the Brain: A Critical Mechanism That Can Advance Therapeutic Development for Ischemic Stroke. Pharmaceutics.

[25] Castillo J., Loza M.I., Mirelman D., Brea J., Blanco M., Sobrino T., Campos F. (2016). A novel mechanism of neuroprotection: Blood glutamate grabber. J. Cereb. Blood Flow Metab..

[26] da Silva-Candal A., Perez-Diaz A., Santamaría M., Correa-Paz C., Rodríguez-Yáñez M., Ardá A., Pérez-Mato M., Iglesias-Rey R., Brea J., Azuaje J. (2018). Clinical validation of blood/brain glutamate grabbing in acute ischemic stroke. Ann. Neurol..

[27] García-Bonilla L., Campos M., Giralt D., Salat D., Chacón P., Guillamon M.M.H., Rosell A., Montaner J. (2012). Evidence for the efficacy of statins in animal stroke models: A meta-analysis. J. Neurochem..

[28] Zhao S.-P., Zhao W., Xiao Z.-J. (2019). The Benefits and Risks of Statin Therapy in Ischemic Stroke: A Review of the Literature. Neurol. India.

[29] Qian Y., Chopp M., Chen J. (2020). Emerging role of microRNAs in ischemic stroke with comorbidities. Exp. Neurol..

[30] Jiang X., Andjelkovic A.V., Zhu L., Yang T., Bennett M.V.L., Chen J., Keep R.F., Shi Y. (2018). Blood-brain barrier dysfunction and recovery after ischemic stroke. Prog. Neurobiol..

[31] Dong X., Gao J., Su Y., Wang Z. (2020). Nanomedicine for Ischemic Stroke. Int. J. Mol. Sci..

[32] Fukuta T., Oku N., Kogure K. (2022). Application and Utility of Liposomal Neuroprotective Agents and Biomimetic Nanoparticles for the Treatment of Ischemic Stroke. Pharmaceutics.

[33] Teleanu D.M., Chircov C., Grumezescu A.M., Teleanu R.I. (2019). Neuronanomedicine: An Up-to-Date Overview. Pharmaceutics.

[34] Durzyńska J., Przysiecka Ł., Nawrot R., Barylski J., Nowicki G., Warowicka A., Musidlak O., Goździcka-Józefiak A. (2015). Viral and Other Cell-Penetrating Peptides as Vectors of Therapeutic Agents in Medicine. J. Pharmacol. Exp. Ther..

[35] Kurrikoff K., Vunk B., Langel Ü. (2021). Status update in the use of cell-penetrating peptides for the delivery of macromolecular therapeutics. Expert Opin. Biol. Ther..

[36] Johnson R.M., Harrison S.D., Maclean D. (2011). Therapeutic Applications of Cell-Penetrating Peptides. Methods Mol. Biol..

[37] Wang F., Cerione R.A., Antonyak M.A. (2021). Isolation and characterization of extracellular vesicles produced by cell lines. STAR Protoc..

[38] Alkaff S.A., Radhakrishnan K., Nedumaran A.M., Liao P., Czarny B. (2020). Nanocarriers for Stroke Therapy: Advances and Obstacles in Translating Animal Studies. Int. J. Nanomed..

[39] Fatima S., Quadri S.N., Parveen S., Beg S., Barkat A., Samim M., Abdin M., Ahmad F.J. (2021). Nanomedicinal Strategies as Emerging Therapeutic Avenues to Treat and Manage Cerebral Ischemia. CNS Neurol. Disord. Drug Targets.

[40] Bernardo-Castro S., Albino I., Barrera-Sandoval Á.M., Tomatis F., Sousa J., Martins E., Simões S., Lino M., Ferreira L., Sargento-Freitas J. (2021). Therapeutic Nanoparticles for the Different Phases of Ischemic Stroke. Life.

[41] He W., Zhang Z., Sha X. (2021). Nanoparticles-mediated emerging approaches for effective treatment of ischemic stroke. Biomaterials.

[42] Ruíz M.A., Clares B., Morales M.E., Gallardo V. (2008). Vesicular Lipidic Systems, Liposomes, PLO, and Liposomes–PLO: Characterization by Electronic Transmission Microscopy. Drug Dev. Ind. Pharm..

[43] Spuch C., Navarro C. (2011). Liposomes for Targeted Delivery of Active Agents against Neurodegenerative Diseases (Alzheimer’s Disease and Parkinson’s Disease). J. Drug Deliv..

[44] Teleanu R.I., Preda M.D., Niculescu A.-G., Vladâcenco O., Radu C.I., Grumezescu A.M., Teleanu D.M. (2022). Current Strategies to Enhance Delivery of Drugs across the Blood–Brain Barrier. Pharmaceutics.

[45] Satapathy M., Yen T.-L., Jan J.-S., Tang R.-D., Wang J.-Y., Taliyan R., Yang C.-H. (2021). Solid Lipid Nanoparticles (SLNs): An Advanced Drug Delivery System Targeting Brain through BBB. Pharmaceutics.

[46] Gastaldi L., Battaglia L., Peira E., Chirio D., Muntoni E., Solazzi I., Gallarate M., Dosio F. (2014). Solid lipid nanoparticles as vehicles of drugs to the brain: Current state of the art. Eur. J. Pharm. Biopharm..

[47] Landowski L.M., Niego B., Sutherland B.A., Hagemeyer C.E., Howells D.W. (2020). Applications of Nanotechnology in the Diagnosis and Therapy of Stroke. Semin. Thromb. Hemost..

[48] Nair R., Kumar A.C., Priya V.K., Yadav C.M., Raju P.Y. (2012). Formulation and evaluation of chitosan solid lipid nanoparticles of carbamazepine. Lipids Health Dis..

[49] Panagiotou S., Esaha S. (2015). Therapeutic benefits of nanoparticles in stroke. Front. Neurosci..

[50] Karatas H., Aktas Y., Gursoy-Ozdemir Y., Bodur E., Yemisci M., Caban S., Vural A., Pinarbasli O., Capan Y., Fernandez-Megia E. (2009). A Nanomedicine Transports a Peptide Caspase-3 Inhibitor across the Blood–Brain Barrier and Provides Neuroprotection. J. Neurosci..

[51] Reddy M.K., Labhasetwar V. (2009). Nanoparticle-mediated delivery of superoxide dismutase to the brain: An effective strategy to reduce ischemia-reperfusion injury. FASEB J..

[52] Ortega E., Blanco S., Ruiz A., Peinado M.A., Peralta S., Morales M.E. (2021). Lipid nanoparticles for the transport of drugs like dopamine through the blood-brain barrier. J. Nanopart. Res..

[53] Liu X., Ye M., An C., Pan L., Ji L. (2013). The effect of cationic albumin-conjugated PEGylated tanshinone IIA nanoparticles on neuronal signal pathways and neuroprotection in cerebral ischemia. Biomaterials.

[54] Blanco S., Peralta S., Morales M.E., Martínez-Lara E., Pedrajas J.R., Castán H., Peinado M., Ruiz M.A. (2020). Hyaluronate Nanoparticles as a Delivery System to Carry Neuroglobin to the Brain after Stroke. Pharmaceutics.

[55] Peinado M., Ovelleiro D., del Moral M.L., Hernández R., Martínez-Lara E., Siles E., Pedrajas J.R., García-Martín M.L., Caro C., Peralta S. (2021). Biological Implications of a Stroke Therapy Based in Neuroglobin Hyaluronate Nanoparticles. Neuroprotective Role and Molecular Bases. Int. J. Mol. Sci..

[56] Peralta S., Blanco S., Hernández R., Castán H., Siles E., Martínez-Lara E., Morales M.E., Peinado M., Ruiz M.A. (2019). Synthesis and characterization of different sodium hyaluronate nanoparticles to transport large neurotherapheutic molecules through blood brain barrier after stroke. Eur. Polym. J..

[57] Burmester T., Weich B., Reinhardt S., Hankeln T. (2000). A vertebrate globin expressed in the brain. Nature.

[58] Exertier C., Montemiglio L.C., Freda I., Gugole E., Parisi G., Savino C., Vallone B. (2022). Neuroglobin, clues to function and mechanism. Mol. Asp. Med..

[59] Keppner A., Maric D., Correia M., Koay T.W., Orlando I.M., Vinogradov S.N., Hoogewijs D. (2020). Lessons from the post-genomic era: Globin diversity beyond oxygen binding and transport. Redox Biol..

[60] Gorabi A.M., Aslani S., Barreto G.E., Báez-Jurado E., Kiaie N., Jamialahmadi T., Sahebkar A. (2020). The potential of mitochondrial modulation by neuroglobin in treatment of neurological disorders. Free Radic. Biol. Med..

[61] Fordel E., Geuens E., Dewilde S., De Coen W., Moens L. (2004). Hypoxia/Ischemia and the Regulation of Neuroglobin and Cytoglobin Expression. IUBMB Life.

[62] Liu N., Yu Z., Gao X., Song Y., Yuan J., Xun Y., Wang T., Yan F., Yuan S., Zhang J. (2016). Establishment of Cell-Based Neuroglobin Promoter Reporter Assay for Neuroprotective Compounds Screening. CNS Neurol. Disord. Drug Targets.

[63] Ren C., Wang P., Wang B., Li N., Li W., Zhang C., Jin K., Ji X. (2015). Limb remote ischemic per-conditioning in combination with post-conditioning reduces brain damage and promotes neuroglobin expression in the rat brain after ischemic stroke. Restor. Neurol. Neurosci..

[64] Schmidt-Kastner R., Haberkamp M., Schmitz C., Hankeln T., Burmester T. (2006). Neuroglobin mRNA expression after transient global brain ischemia and prolonged hypoxia in cell culture. Brain Res..

[65] Shang A., Zhou D., Wang L., Gao Y., Fan M., Wang X., Zhou R., Zhang C. (2006). Increased neuroglobin levels in the cerebral cortex and serum after ischemia–reperfusion insults. Brain Res..

[66] Song X., Xu R., Xie F., Zhu H., Zhu J., Wang X. (2014). Hemin offers neuroprotection through inducing exogenous neuroglobin in focal cerebral hypoxic-ischemia in rats. Int. J. Clin. Exp. Pathol..

[67] Pandya R.S., Mao L., Zhou H., Zhou S., Zeng J., Popp A.J., Wang X. (2011). Central nervous system agents for ischemic stroke: Neuroprotection mechanisms. Central Nerv. Syst. Agents Med. Chem..

[68] Sun Y., Jin K., Mao X.O., Zhu Y., Greenberg D.A. (2001). Neuroglobin is up-regulated by and protects neurons from hypoxic-ischemic injury. Proc. Natl. Acad. Sci. USA.

[69] Jin K., Mao X., Xie L., Greenberg D.A. (2011). Neuroglobin Expression in Human Arteriovenous Malformation and Intracerebral Hemorrhage. Acta Neurochir. Suppl..

[70] Jin K., Mao Y., Mao X., Xie L., Greenberg D.A. (2010). Neuroglobin Expression in Ischemic Stroke. Stroke.

[71] Fiocchetti M., Cipolletti M., Brandi V., Polticelli F., Ascenzi P. (2017). Neuroglobin and friends. J. Mol. Recognit..

[72] Raida Z., Hundahl C.A., Nyengaard J.R., Hay-Schmidt A. (2013). Neuroglobin Over Expressing Mice: Expression Pattern and Effect on Brain Ischemic Infarct Size. PLoS ONE.

[73] Hundahl C., Kelsen J., Kjær K., Rønn L.C.B., Weber R.E., Geuens E., Hay-Schmidt A., Nyengaard J.R. (2006). Does neuroglobin protect neurons from ischemic insult? A quantitative investigation of neuroglobin expression following transient MCAo in spontaneously hypertensive rats. Brain Res..

[74] Raida Z., Hundahl C.A., Kelsen J., Nyengaard J.R., Hay-Schmidt A. (2012). Reduced infarct size in neuroglobin-null mice after experimental stroke in vivo. Exp. Transl. Stroke Med..

[75] Shang A., Liu K., Wang H., Wang J., Hang X., Yang Y., Wang Z., Zhang C., Zhou D. (2011). Neuroprotective effects of neuroglobin after mechanical injury. Neurol. Sci..

[76] Khan A.A., Wang Y., Sun Y., Mao X.O., Xie L., Miles E., Graboski J., Chen S., Ellerby L.M., Jin K. (2006). Neuroglobin-overexpressing transgenic mice are resistant to cerebral and myocardial ischemia. Proc. Natl. Acad. Sci. USA.

[77] Wang J.-Y., Shen J., Gao Q., Ye Z.-G., Yang S.-Y., Liang H.-W., Bruce I.C., Luo B.-Y., Xia Q. (2008). Ischemic Postconditioning Protects Against Global Cerebral Ischemia/Reperfusion-Induced Injury in Rats. Stroke.

[78] Raychaudhuri S., Skommer J., Henty K., Birch N., Brittain T. (2010). Neuroglobin protects nerve cells from apoptosis by inhibiting the intrinsic pathway of cell death. Apoptosis.

[79] Sun Y., Jin K., Peel A., Mao X.O., Xie L., Greenberg D.A. (2003). Neuroglobin protects the brain from experimental stroke in vivo. Proc. Natl. Acad. Sci. USA.

[80] Greenberg D.A., Jin K., Khan A.A. (2008). Neuroglobin: An endogenous neuroprotectant. Curr. Opin. Pharmacol..

[81] Yu Z., Fan X., Lo E.H., Wang X. (2009). Neuroprotective roles and mechanisms of neuroglobin. Neurol. Res..

[82] Zhu Y., Sun Y., Jin K., Greenberg D.A. (2002). Hemin induces neuroglobin expression in neural cells. Blood.

[83] Avila-Rodríguez M., Garcia-Segura L.M., Lanussa O.A.H., Baez E., Gonzalez J., Barreto G.E. (2016). Tibolone protects astrocytic cells from glucose deprivation through a mechanism involving estrogen receptor beta and the upregulation of neuroglobin expression. Mol. Cell. Endocrinol..

[84] Lanussa O.A.H., Avila-Rodríguez M., Baez-Jurado E., Zamudio-Rodriguez J.A., Echeverria V., Garcia-Segura L.M., Barreto G.E. (2018). Tibolone Reduces Oxidative Damage and Inflammation in Microglia Stimulated with Palmitic Acid through Mechanisms Involving Estrogen Receptor Beta. Mol. Neurobiol..

[85] Barreto G., McGovern A., Garcia-Segura L. (2021). Role of Neuroglobin in the Neuroprotective Actions of Estradiol and Estrogenic Compounds. Cells.

[86] Acaz-Fonseca E., Castelló-Ruiz M., Burguete M.C., Aliena-Valero A., Salom J.B., Torregrosa G., García-Segura L.M. (2020). Insight into the molecular sex dimorphism of ischaemic stroke in rat cerebral cortex: Focus on neuroglobin, sex steroids and autophagy. Eur. J. Neurosci..

[87] Baez E., Echeverria V., Cabezas R., Ávila-Rodriguez M., Garcia-Segura L.M., Barreto G.E. (2016). Protection by Neuroglobin Expression in Brain Pathologies. Front. Neurol..

[88] Kakar S., Hoffman F.G., Storz J.F., Fabian M., Hargrove M.S. (2010). Structure and reactivity of hexacoordinate hemoglobins. Biophys. Chem..

[89] Liu J., Yu Z., Guo S., Lee S.-R., Xing C., Zhang C., Gao Y., Nicholls D.G., Lo E.H., Wang X. (2009). Effects of neuroglobin overexpression on mitochondrial function and oxidative stress following hypoxia/reoxygenation in cultured neurons. J. Neurosci. Res..

[90] Tejero J., Sparacino-Watkins C.E., Ragireddy V., Frizzell S., Gladwin M.T. (2015). Exploring the Mechanisms of the Reductase Activity of Neuroglobin by Site-Directed Mutagenesis of the Heme Distal Pocket. Biochemistry.

[91] Brunori M., Giuffrè A., Nienhaus K., Nienhaus G.U., Scandurra F.M., Vallone B. (2005). Neuroglobin, nitric oxide, and oxygen: Functional pathways and conformational changes. Proc. Natl. Acad. Sci. USA.

[92] Van Doorslaer S., Dewilde S., Kiger L., Nistor S.V., Goovaerts E., Marden M.C., Moens L. (2003). Nitric oxide binding properties of neuroglobin: A characterization by EPR and flash photolysis. J. Biol. Chem..

[93] Hundahl C.A., Allen G.C., Nyengaard J.R., Dewilde S., Carter B.D., Kelsen J., Hay-Schmidt A. (2008). Neuroglobin in the Rat Brain: Localization. Neuroendocrinology.

[94] Mammen P.P., Shelton J.M., Goetsch S.C., Williams S.C., Richardson J.A., Garry M.G., Garry D.J. (2002). Neuroglobin, A Novel Member of the Globin Family, Is Expressed in Focal Regions of the Brain. J. Histochem. Cytochem..

[95] Eliasson M.J.L., Huang Z., Ferrante R.J., Sasamata M., Molliver M.E., Snyder S.H., Moskowitz M.A. (1999). Neuronal Nitric Oxide Synthase Activation and Peroxynitrite Formation in Ischemic Stroke Linked to Neural Damage. J. Neurosci..

[96] Herold S., Fago A. (2005). Reactions of peroxynitrite with globin proteins and their possible physiological role. Comp. Biochem. Physiol. Part A Mol. Integr. Physiol..

[97] Nicolis S., Monzani E., Ciaccio C., Ascenzi P., Moens L., Casella L. (2007). Reactivity and endogenous modification by nitrite and hydrogen peroxide: Does human neuroglobin act only as a scavenger?. Biochem. J..

[98] Yu Z., Zhang Y., Liu N., Yuan J., Lin L., Zhuge Q., Xiao J., Wang X. (2016). Roles of Neuroglobin Binding to Mitochondrial Complex III Subunit Cytochrome c1 in Oxygen-Glucose Deprivation-Induced Neurotoxicity in Primary Neurons. Mol. Neurobiol..

[99] Chen L.-M., Xiong Y.-S., Kong F.-L., Qu M., Wang Q., Chen X.-Q., Wang J.-Z., Zhu L.-Q. (2012). Neuroglobin attenuates Alzheimer-like tau hyperphosphorylation by activating Akt signaling. J. Neurochem..

[100] Lee H.M., Greeley G.H., Englander E.W. (2011). Transgenic overexpression of neuroglobin attenuates formation of smoke-inhalation-induced oxidative DNA damage, in vivo, in the mouse brain. Free Radic. Biol. Med..

[101] Antao S.T., Duong T.H., Aran R., Witting P.K. (2010). Neuroglobin Overexpression in Cultured Human Neuronal Cells Protects Against Hydrogen Peroxide Insult via Activating Phosphoinositide-3 Kinase and Opening the Mitochondrial K_ATP_ Channel. Antioxid. Redox Signal..

[102] Cai B., Li W., Mao X., Winters A., Ryou M.-G., Liu R., Greenberg D.A., Wang N., Jin K., Yang S.-H. (2016). Neuroglobin Overexpression Inhibits AMPK Signaling and Promotes Cell Anabolism. Mol. Neurobiol..

[103] Zhang C., Hao X., Chang J., Geng Z., Wang Z. (2019). Mn-TAT PTD-Ngb attenuates oxidative injury by an enhanced ROS scavenging ability and the regulation of redox signaling pathway. Sci. Rep..

[104] Zhang B., Ji X., Zhang S., Ren H., Wang M., Guo C., Li Y. (2013). Hemin-mediated neuroglobin induction exerts neuroprotection following ischemic brain injury through PI3K/Akt signaling. Mol. Med. Rep..

[105] Zhang B., Liu Y., Li Y., Zhe X., Zhang S., Zhang L. (2018). Neuroglobin promotes the proliferation and suppresses the apoptosis of glioma cells by activating the PI3K/AKT pathway. Mol. Med. Rep..

[106] Yu Z., Liu N., Li Y., Xu J., Wang X. (2013). Neuroglobin overexpression inhibits oxygen–glucose deprivation-induced mitochondrial permeability transition pore opening in primary cultured mouse cortical neurons. Neurobiol. Dis..

[107] Fago A., Mathews A.J., Moens L., Dewilde S., Brittain T. (2006). The reaction of neuroglobin with potential redox protein partners cytochrome *b*_5_ and cytochrome *c*. FEBS Lett..

[108] Duong T.T.H., Witting P.K., Antao S.T., Parry S.N., Kennerson M., Lai B., Vogt S., Lay P.A., Harris H.H. (2009). Multiple protective activities of neuroglobin in cultured neuronal cells exposed to hypoxia re-oxygenation injury. J. Neurochem..

[109] Goldsmith K.C., Gross M., Peirce S., Luyindula D., Liu X., Vu A., Sliozberg M., Guo R., Zhao H., Reynolds C.P. (2012). Mitochondrial Bcl-2 Family Dynamics Define Therapy Response and Resistance in Neuroblastoma. Cancer Res..

[110] Boldogh I.R., Pon L.A. (2006). Interactions of mitochondria with the actin cytoskeleton. Biochim. Biophys. Acta.

[111] Yu Z., Cheng C., Liu Y., Liu N., Lo E.H., Wang X. (2018). Neuroglobin promotes neurogenesis through Wnt signaling pathway. Cell Death Dis..

[112] Wen H., Liu L., Zhan L., Liang D., Li L., Liu D., Sun W., Xu E. (2018). Neuroglobin mediates neuroprotection of hypoxic postconditioning against transient global cerebral ischemia in rats through preserving the activity of Na^+^/K^+^ ATPases. Cell Death Dis..

[113] Li L., Liu Q.R., Xiong X.X., Liu J.M., Lai X.J., Cheng C., Pan F., Chen Y., Bin Yu S., Yu A.C.H. (2014). Neuroglobin Promotes Neurite Outgrowth via Differential Binding to PTEN and Akt. Mol. Neurobiol..

[114] Chen X., Liu Y., Zhang L., Zhu P., Zhu H., Yang Y., Guan P. (2015). Long-term neuroglobin expression of human astrocytes following brain trauma. Neurosci. Lett..

[115] Hernández R., Blanco S., Peragón J., Pedrosa J., Peinado M. (2013). Hypobaric Hypoxia and Reoxygenation Induce Proteomic Profile Changes in the Rat Brain Cortex. Neuromol. Med..

[116] Ovelleiro D., Blanco S., Hernández R., Peinado M. (2017). Comparative proteomic study of early hypoxic response in the cerebral cortex of rats submitted to two different hypoxic models. Proteom. Clin. Appl..

[117] Peinado M., Hernández R., Peragón J., Ovelleiro D., Pedrosa J., Blanco S. (2014). Proteomic characterization of nitrated cell targets after hypobaric hypoxia and reoxygenation in rat brain. J. Proteom..

[118] Haines B.A., Davis D.A., Zykovich A., Peng B., Rao R., Mooney S.D., Jin K., Greenberg D.A. (2012). Comparative protein interactomics of neuroglobin and myoglobin. J. Neurochem..

[119] Lin L., Li X., Pan C., Lin W., Shao R., Liu Y., Zhang J., Luo Y., Qian K., Shi M. (2019). ATXN2L upregulated by epidermal growth factor promotes gastric cancer cell invasiveness and oxaliplatin resistance. Cell Death Dis..

[120] Peroni D., Negro A., Bähr M., Dietz G.P. (2007). Intracellular delivery of Neuroglobin using HIV-1 TAT protein transduction domain fails to protect against oxygen and glucose deprivation. Neurosci. Lett..

[121] Zhou G.-Y., Zhou S.-N., Lou Z.-Y., Zhu C.-S., Zheng X.-P., Hu X.-Q. (2008). Translocation and neuroprotective properties of transactivator-of-transcription protein-transduction domain–neuroglobin fusion protein in primary cultured cortical neurons. Biotechnol. Appl. Biochem..

[122] Belayev L., Busto R., Zhao W., Ginsberg M.D. (1996). Quantitative evaluation of blood-brain barrier permeability following middle cerebral artery occlusion in rats. Brain Res..

[123] Rosenberg G.A., Estrada E.Y., Dencoff J.E. (1998). Matrix Metalloproteinases and TIMPs Are Associated with Blood-Brain Barrier Opening After Reperfusion in Rat Brain. Stroke.

[124] McColl B.W., Rothwell N.J., Allan S. (2008). Systemic Inflammation Alters the Kinetics of Cerebrovascular Tight Junction Disruption after Experimental Stroke in Mice. J. Neurosci..

[125] Cai B., Lin Y., Xue X.-H., Fang L., Wang N., Wu Z.-Y. (2011). TAT-mediated delivery of neuroglobin protects against focal cerebral ischemia in mice. Exp. Neurol..

[126] Fiocchetti M., De Marinis E., Ascenzi P., Marino M. (2013). Neuroglobin and neuronal cell survival. Biochim. Biophys. Acta Proteins Proteom..

[127] Yu Z., Liu N., Liu J., Yang K., Wang X. (2012). Neuroglobin, a Novel Target for Endogenous Neuroprotection against Stroke and Neurodegenerative Disorders. Int. J. Mol. Sci..

[128] Dietz G.P.H., Bähr M. (2004). Delivery of bioactive molecules into the cell: The Trojan horse approach. Mol. Cell. Neurosci..

[129] Luyckx E., Van Acker Z.P., Ponsaerts P., Dewilde S. (2019). Neuroglobin Expression Models as a Tool to Study Its Function. Oxidative Med. Cell. Longev..

[130] Hanson L.R., Frey W.H. (2008). Intranasal delivery bypasses the blood-brain barrier to target therapeutic agents to the central nervous system and treat neurodegenerative disease. BMC Neurosci..

[131] Monge-Fuentes V., Mayer A.B., Lima M.R., Geraldes L.R., Zanotto L.N., Moreira K.G., Martins O.P., Piva H.L., Felipe M.S.S., Amaral A.C. (2021). Dopamine-loaded nanoparticle systems circumvent the blood–brain barrier restoring motor function in mouse model for Parkinson’s Disease. Sci. Rep..

[132] Fortuni B., Inose T., Ricci M., Fujita Y., Van Zundert I., Masuhara A., Fron E., Mizuno H., Latterini L., Rocha S. (2019). Polymeric Engineering of Nanoparticles for Highly Efficient Multifunctional Drug Delivery Systems. Sci. Rep..

[133] Ahlawat J., Henriquez G., Narayan M. (2018). Enhancing the Delivery of Chemotherapeutics: Role of Biodegradable Polymeric Nanoparticles. Molecules.

[134] Cortés H., Alcalá-Alcalá S., Caballero-Florán I.H., Bernal-Chávez S.A., Ávalos-Fuentes A., González-Torres M., Carmen M.G.-D., Figueroa-González G., Reyes-Hernández O.D., Floran B. (2020). A Reevaluation of Chitosan-Decorated Nanoparticles to Cross the Blood-Brain Barrier. Membranes.

[135] Jensen G., Holloway J.L., Stabenfeldt S.E. (2020). Hyaluronic Acid Biomaterials for Central Nervous System Regenerative Medicine. Cells.

[136] Sawada R., Nakano-Doi A., Matsuyama T., Nakagomi N., Nakagomi T.T. (2020). CD44 expression in stem cells and niche microglia/macrophages following ischemic stroke. Stem Cell Investig..

[137] Donahue N.D., Acar H., Wilhelm S. (2019). Concepts of nanoparticle cellular uptake, intracellular trafficking, and kinetics in nanomedicine. Adv. Drug Deliv. Rev..

[138] Varkouhi A.K., Scholte M., Storm G., Haisma H.J. (2011). Endosomal escape pathways for delivery of biologicals. J. Control. Release.

[139] Pei D., Buyanova M. (2019). Overcoming Endosomal Entrapment in Drug Delivery. Bioconj. Chem..

[140] Correa-Paz C., da Silva-Candal A., Polo E., Parcq J., Vivien D., Maysinger D., Pelaz B., Campos F. (2021). New Approaches in Nanomedicine for Ischemic Stroke. Pharmaceutics.

